# Assessment of Knowledge and Clinical Practice Patterns in Restorative Material Selection for Anterior Teeth Among Dental Practitioners

**DOI:** 10.7759/cureus.112948

**Published:** 2026-07-19

**Authors:** Sameer Chauhan, Bhavik Ramanlal Patel, Akshat A Chokshi, Amandeep Kaur, Ashfaq Yaqoob, Deepika Singla, Afroz K Syed

**Affiliations:** 1 Department of Prosthodontics, K.M. Shah Dental College and Hospital, Sumandeep Vidyapeeth (Deemed to be University), Vadodara, IND; 2 Department of Orthodontics, A.J. Institute of Dental Sciences, Mangalore, IND; 3 Department of Dentistry, Taunton Village Dental, Oshawa, CAN; 4 Department of Oral Health Sciences, Postgraduate Institute of Medical Education and Research Satellite Centre, Sangrur, IND; 5 Department of Prosthodontics, King Khalid University Hospital, Abha, SAU; 6 Department of Conservative Dentistry and Endodontics, Desh Bhagat Dental College and Hospital, Mandi Gobindgarh, IND; 7 Department of Dentistry, Anne Yarlagadda Charitable Trust, Vijayawada, IND

**Keywords:** anterior teeth, clinical competence, composite resins, dental restoration, questionnaire, surveys

## Abstract

Introduction: The selection of restorative materials for anterior teeth is a critical aspect of contemporary restorative dentistry that requires the integration of esthetic, functional, and biological considerations. The increasing availability of restorative materials and adhesive technologies necessitates evidence-based decision-making to optimize treatment outcomes. However, variations in practitioner knowledge and clinical practice may influence restorative material selection and treatment success. Therefore, this study aimed to assess the knowledge and clinical practice patterns of dental practitioners regarding restorative material selection for anterior teeth and to identify factors associated with evidence-based restorative decision-making.

Materials and methods: A cross-sectional questionnaire-based survey was conducted with 160 registered dental practitioners, including non-specialist and specialist dental practitioners. A structured, self-administered questionnaire consisting of demographic details, 10 knowledge-based questions, and 10 practice-based questions was developed following a literature review and expert validation. A pilot study was conducted with 30 practitioners to assess the clarity and reliability. Knowledge and practice scores were calculated using predefined criteria. Data were analyzed using descriptive and inferential statistics including independent t-tests, chi-squared tests, Pearson's correlation analysis, and binary logistic regression. Statistical significance was set at p<0.05.

Results: Among the 160 participants, specialists demonstrated significantly higher knowledge scores (7.88±2.01) and practice scores (7.59±2.06) than non-specialists (5.92±2.03 and 5.41±2.12, respectively; p<0.001). Good knowledge was observed in 56 (35%) participants, while good clinical practice was observed in 41 (25.6%). Knowledge scores showed a strong positive correlation with practice scores (r=0.73; p=0.002). Years of clinical experience was positively associated with both knowledge and practice. Logistic regression analysis identified specialist qualifications, greater clinical experience, and higher knowledge scores as significant predictors of good clinical practice (p<0.05).

Conclusion: Specialist dental practitioners demonstrated superior knowledge, clinical practice patterns, and overall competency in restorative material selection for anterior teeth compared with non-specialists. Knowledge, professional qualifications, and clinical experience are significant determinants of evidence-based restorative decision-making. Continuous professional education and training programs may enhance restorative treatment planning and improve clinical outcomes.

## Introduction

Restoration of the anterior teeth is one of the most demanding aspects of contemporary restorative dentistry, as it requires successful integration of esthetics, function, durability, and preservation of tooth structure [1]. Advances in adhesive dentistry and dental biomaterials have expanded the range of restorative options available to clinicians, including direct composite restorations, ceramic veneers, and full-coverage ceramic crowns [2]. The selection of an appropriate restorative material is influenced by several factors, including the extent of tooth destruction, esthetic requirements, functional demands, material properties, patient expectations, economic considerations, and clinician knowledge and experience [3]. Appropriate material selection is critical for achieving long-term clinical success, patient satisfaction, and the preservation of natural tooth tissues.

Despite the availability of evidence-based guidelines and continuous innovations in restorative materials, variations in clinical decision-making among dental practitioners remain common. Differences in educational background, specialty training, years of professional experience, and exposure to continuing dental education may significantly influence restorative material choices [4]. Inappropriate selection of restorative materials may compromise esthetic outcomes, longevity of restorations, and overall treatment success. Therefore, assessing the level of knowledge and clinical practice among dental practitioners is essential for identifying potential gaps between contemporary scientific evidence and routine clinical practice [5].

This study aimed to evaluate the knowledge and clinical practice patterns of dental practitioners regarding restorative material selection for anterior teeth. Specifically, the objectives were to assess practitioners' knowledge of evidence-based restorative material selection and their routine clinical practice patterns, compare these outcomes between general dental practitioners and specialists, examine the relationship between knowledge and clinical practice, and identify demographic and professional factors associated with evidence-based restorative decision-making.

## Materials and methods

Study design and setting

A cross-sectional questionnaire-based survey was conducted at the Department of Conservative Dentistry and Endodontics of Desh Bhagat Dental College and Hospital in Mandi Gobindgarh, Punjab, India. Registered dental practitioners practicing in Punjab, India, were recruited through professional dental associations, email networks, and social media platforms. The study was conducted over a period of two months, from August 2025 to September 2025, following approval from the Institutional Ethical Committee of Desh Bhagat Dental College and Hospital (approval number: DBDCH/IEC/2025/05/032; date: May 14, 2025).

Study population and eligibility criteria

The study population comprised registered dental practitioners, including general dental practitioners and dental specialists who actively engaged in restorative dental procedures. The specialist group comprised multiple dental specialties with potentially different levels of expertise in restorative dentistry. However, subgroup analyses according to specialty were not performed because of the limited sample size within individual specialties. Dentists with valid registration with the respective State Dental Council or the Dental Council of India and willing to provide informed consent were eligible for participation. Undergraduate students, interns, dentists not involved in restorative treatment procedures, and participants who submitted incomplete questionnaires were excluded from the study.

Sample size estimation

The sample size was calculated using the formula for estimating a single population proportion $n = \frac{Z^2 P(1-P)}{d^2}$, where Z was set at 1.96 for a 95% confidence level, P was assumed to be 50% because of the absence of robust prevalence data regarding adequate knowledge of restorative material selection among dental practitioners, and d was fixed at 8% precision [6]. A minimum sample size of 150 participants was required for this study. To compensate for an anticipated non-response rate of approximately 10%, the target sample size was increased to 165 participants. However, 160 complete responses were ultimately obtained and included in the final analysis. The sample size estimation was cross-validated using G*Power Version 3.1.9.7 (Heinrich Heine University Düsseldorf, Düsseldorf, Germany), assuming an 80% statistical power and a significance level of 5%.

Sampling technique

A convenience sampling strategy was employed in this study. The questionnaires were distributed through professional dental associations, social media groups, email networks, and direct communication with dental practitioners. Participation was voluntary, and no incentives were provided. The online survey remained open for a predefined period, during which periodic reminders were circulated to improve the response rate.

Questionnaire development

A structured self-administered questionnaire was specifically developed for the present study after an extensive review of contemporary literature, evidence-based restorative dentistry guidelines, and previously published surveys evaluating restorative material selection among dental professionals. The questionnaire was prepared by a multidisciplinary team of specialists in conservative dentistry and endodontics, prosthodontics, and public dental health with expertise in restorative treatment planning and survey research methodology.

The final questionnaire consisted of three sections. The first section collected demographic and professional information including age, sex, qualifications, specialty, years of clinical experience, and type of practice. The second section comprised 10 knowledge-based multiple-choice questions assessing participants' understanding of indications and contraindications of restorative materials, conservative treatment approaches, material properties, esthetic considerations, shade selection principles, and factors influencing restoration longevity. The third section contained 10 practice-based questions designed to evaluate routine clinical decision-making, restorative material preferences, isolation techniques, shade selection practices, patient communication, and evidence-based treatment planning (see Appendices). 

Content validity and face validity

Content validity was established through expert review by a panel of five subject experts: prosthodontists, conservative dentists, and dental public health specialists. Each expert independently evaluated the questionnaire for relevance, clarity, comprehensiveness, and appropriateness of individual items. The recommendations provided by the expert panel were incorporated to improve the questionnaire's wording, structure, and content coverage. Facial validity was assessed by a small group of practicing dentists to ensure that all the questions were understandable, unambiguous, and clinically relevant.

Pilot study

A pilot study was conducted among 30 dental practitioners who fulfilled the eligibility criteria, but were not included in the final analysis. The purpose of the pilot study was to evaluate the clarity, feasibility, completion time, comprehensibility, and response consistency of the questionnaire. The feedback obtained during the pilot phase resulted in minor modifications to the wording and sequencing of the selected questions to enhance participant understanding and improve survey flow.

Reliability assessment

The internal consistency and reliability of the questionnaire were assessed using Cronbach's alpha. The responses obtained during the pilot study were used for reliability testing. A Cronbach's alpha value of 0.70 or greater was considered indicative of acceptable internal consistency. The final questionnaire demonstrated satisfactory reliability, supporting its suitability for use with the main study population.

Questionnaire scoring

Knowledge was evaluated using 10 multiple-choice questions. Each correct response received 1 point, whereas incorrect responses received 0 points. Consequently, the total knowledge score ranged from 0 to 10. Participants scoring 0-4 were categorized as having poor knowledge, 5-7 as moderate knowledge, and 8-10 as good knowledge. Clinical practice patterns were assessed using 10 evidence-based practice questions. Responses consistent with contemporary restorative dentistry recommendations were assigned 1 point, while non-evidence-based responses received 0 points. The total practice score ranged from 0 to 10. Scores of 0-4 indicated poor practice, scores of 5-7 indicated moderate practice, and scores of 8-10 reflected good practice. The overall competency score was calculated by combining the knowledge and practice scores, resulting in a total possible score ranging from 0 to 20. Participants were categorized as having poor overall competency (0-9), moderate overall competency (10-14), or good overall competency (15-20) (Table 1).

**Table 1:** Scoring for questionnaire responses

Grade	Knowledge score (0-10)	Practice score (0-10)	Total score (0-20)
Poor	0-4	0-4	0-9
Moderate	5-7	5-7	10-14
Good	8-10	8-10	15-20

Data collection procedure

The final questionnaire was converted into an electronic survey using Google Forms (Google LLC, Mountain View, California, United States). The first page contained participant information, study objectives, confidentiality assurance, and an informed consent form. Participants were permitted to proceed only after providing their electronic consent. All responses were collected anonymously, and no personally identifiable information was recorded. Data collection continued until the required sample size was reached.

Outcome measures

The primary outcome measure was the knowledge score regarding restorative material selection for anterior tooth restorations. Secondary outcome measures included practice scores, overall competency scores, comparisons between general dental practitioners and specialists, and the identification of factors associated with evidence-based restorative material selection.

Statistical analysis

Data were subsequently analyzed using IBM SPSS Statistics for Windows, Version 25.0 (IBM Corp., Armonk, New York, United States). Descriptive statistics are presented as frequencies, percentages, means, and standard deviations. The normality of continuous variables was assessed using the Shapiro-Wilk test. Comparisons between general dental practitioners and specialists were performed using the independent samples t-test for normally distributed variables or the Mann-Whitney U test for non-normally distributed variables. Associations between categorical variables were evaluated using the chi-squared test or Fisher's exact test, as appropriate. Pearson's correlation coefficient was used to assess the relationships between the knowledge scores, practice scores, and years of clinical experience. Binary logistic regression analysis was performed to identify independent predictors of good clinical practice. Adjusted odds ratios and 95% confidence intervals were calculated. Effect sizes were calculated using Cohen's d for continuous variables and Cramer's V for categorical variables. Statistical significance was set at a p-value of less than 0.05.

## Results

A total of 160 dental practitioners participated in the study, including 85 (53.1%) non-specialist dental practitioners (NSDPs) and 75 (46.9%) specialist dental practitioners (SDPs). All the questionnaires were completed and included in the final analysis.

Demographic and professional characteristics

The demographic and professional characteristics of the participants are summarized in Table 2. The majority of respondents belonged to the 30-40-year age group, followed by those younger than 30 years. Females constituted 86 (53.7%) participants, while males accounted for 74 (46.3%). Among NSDPs, the largest proportion were younger than 30 years, whereas among SDPs, most participants were aged 30-40 years. Significant differences were observed between NSDPs and SDPs in terms of age distribution (p=0.001), years of clinical experience (p=0.002), and type of practice (p=0.005). Practitioners with less than five years of experience were predominantly represented among NSDPs, whereas practitioners with 11-20 years of experience were more common among SDPs. No statistically significant difference was found in sex distribution between the groups (p=0.678).

**Table 2:** Demographic and professional characteristics of the study participants according to qualification (N=160) Data are presented as frequency (n) and percentage (%). Comparisons between NSDPs and SDPs were performed using the chi-squared test. Effect size was calculated using Cramer's V. A p-value of <0.05 was considered statistically significant. *Statistically significant difference (p<0.05). NSDP: non-specialist dental practitioner; SDP: specialist dental practitioner

Characteristic	Category	Total (N=160) n (%)	NSDP (n=85) n (%)	SDP (n=75) n (%)	Chi statistics	P-value	Effect size
Age group	<30 years	62 (38.8)	44 (51.8)	18 (24)	21.45	0.001*	0.31
	30-40 years	68 (42.5)	31 (36.5)	37 (49.3)			
	41-50 years	22 (13.8)	8 (9.4)	14 (18.7)			
	>50 years	8 (5)	2 (2.4)	6 (8)			
Sex	Male	74 (46.3)	38 (44.7)	36 (48)	3.42	0.678	0.03
	Female	86 (53.7)	47 (55.3)	39 (52)			
Years of experience	<5 years	54 (33.8)	38 (44.7)	16 (21.3)	18.70	0.002*	0.29
	5-10 years	52 (32.5)	26 (30.6)	26 (34.7)			
	11-20 years	38 (23.8)	15 (17.6)	23 (30.7)			
	>20 years	16 (10)	6 (7.1)	10 (13.3)			
Practice type	Private practice	72 (45)	48 (56.5)	24 (32)	15.62	0.005*	0.29
	Academic institution	36 (22.4)	14 (16.5)	22 (29.3)			
	Hospital-based	26 (16.3)	12 (14.1)	14 (18.7)			
	Both academic and private	26 (16.3)	11 (12.9)	15 (20)			

Comparison of knowledge and practice scores

A comparison of the knowledge, practice, and overall competency scores between NSDPs and SDPs is presented in Table 3. The overall mean knowledge score was 6.84±2.24, while the mean practice score was 6.43±2.33. Specialists demonstrated significantly higher knowledge scores (7.88±2.01) than non-specialists (5.92±2.03) (p<0.001). Similarly, the mean practice score was significantly higher among SDPs (7.59±2.06) than among NSDPs (5.41±2.12) (p<0.001). The mean overall competency score was 15.47±3.68 among SDPs compared with 11.33±3.89 among NSDPs (p<0.001).

**Table 3:** Comparison of knowledge and practice scores between NSDPs and SDPs Data are presented as mean±standard deviation (SD). Comparisons between groups were performed using the independent samples t-test. Effect size was calculated using Cohen's d. A p-value of <0.05 was considered statistically significant. *Statistically significant difference (p<0.05). NSDP: non-specialist dental practitioner; SDP: specialist dental practitioner

Variable	Total (N=160) mean±SD	NSDP (n=85) mean±SD	SDP (n=75) mean±SD	t-statistic	P-value	Cohen's d
Knowledge score (0-10)	6.84±2.24	5.92±2.03	7.88±2.01	6.19	<0.001*	0.97
Practice score (0-10)	6.43±2.33	5.41±2.12	7.59±2.06	6.66	<0.001*	1.05
Total score (0-20)	13.27±4.30	11.33±3.89	15.47±3.68	6.96	<0.001*	1.10

Distribution of knowledge, practice, and overall competency levels

The distribution of participants according to knowledge, practice, and overall competency categories is presented in Table 4. Good, moderate, and poor knowledge levels were observed in 56 (35%), 72 (45%), and 32 (20%) of the participants, respectively. Among the SDPs, 41 (54.7%) demonstrated good knowledge compared to only 15 (17.6%) NSDPs (p<0.001). With regard to clinical practice, 41 (25.6%) participants exhibited good practice, 74 (46.3%) demonstrated moderate practice, and 45 (28.1%) demonstrated poor practice. Good practice was significantly more frequent among SDPs (31, 41.3%) than among NSDPs (10, 11.8%) (p<0.001). Regarding overall competencies, 52 (32.5%), 74 (46.3%), and 34 (21.2%) participants demonstrated good, moderate, and poor competencies, respectively. Good overall competency was observed among 36 (48%) SDPs compared with 16 (18.9%) NSDPs. Significant associations were identified between qualification status and all three competency domains (p<0.001).

**Table 4:** Distribution of knowledge, practice, and overall competency levels according to qualification Data are presented as frequency (n) and percentage (%). Associations between qualification and competency categories were analyzed using the chi-squared test. Effect size was calculated using Cramer's V. A p-value of <0.05 was considered statistically significant. *Statistically significant association (p<0.05). NSDP: non-specialist dental practitioner; SDP: specialist dental practitioner

Variable	Category (score)	Total (N=160) n (%)	NSDP (n=85) n (%)	SDP (n=75) n (%)	χ² statistic	P-value	Effect size
Knowledge level	Poor (<50%)	32 (20)	27 (31.8)	5 (6.7)	27.8	<0.001*	0.42
	Moderate (50-75%)	72 (45)	43 (50.6)	29 (38.7)			
	Good (>75%)	56 (35)	15 (17.6)	41 (54.7)			
Practice level	Poor (<50%)	45 (28.1)	36 (42.4)	9 (12)	37.8	<0.001*	0.49
	Moderate (50-75%)	74 (46.3)	39 (45.9)	35 (46.7)			
	Good (>75%)	41 (25.6)	10 (11.8)	31 (41.3)			
Overall competency	Poor	34 (21.2)	28 (32.9)	6 (8)	30.5	<0.001*	0.44
	Moderate	74 (46.3)	41 (48.2)	33 (44)			
	Good	52 (32.5)	16 (18.9)	36 (48)			

Correlation analysis

Correlation analysis revealed significant positive relationships between the knowledge scores, practice scores, and years of clinical experience (Table 5). Knowledge scores were strongly correlated with practice scores (r=0.73; p=0.002), indicating that practitioners with higher knowledge levels are more likely to demonstrate evidence-based clinical practice. Years of clinical experience showed moderate positive correlations with knowledge (r=0.42; p=0.004) and practice scores (r=0.51; p=0.034), suggesting that greater professional experience contributes to improved knowledge and clinical performance.

**Table 5:** Correlation between knowledge score, practice score, and years of clinical experience Data are presented as Pearson's correlation coefficient (r). Correlations were analyzed using Pearson's correlation test. A p-value of <0.05 was considered statistically significant. *Statistically significant correlation (p<0.05). r: Pearson's correlation coefficient

Variable	Knowledge score (N=160)		Practice score (N=160)		Years of experience (N=160)	
	r value	P-value	r value	P-value	r value	P-value
Knowledge score	1.00	0.000*	0.73	0.002*	0.42	0.004*
Practice score*	0.73	0.002*	1.00	0.000*	0.51	0.034*
Years of experience	0.42	0.004*	0.51	0.034*	1.00	0.000*

Predictors of good clinical practice

The results of the binary logistic regression analysis are presented in Table 6. Specialist qualification was a significant predictor of good clinical practice, with specialists exhibiting approximately fivefold greater odds of achieving good practice scores than NSDPs (p<0.001). Practitioners with >10 years of clinical experience were 2.41 times more likely to demonstrate good clinical practice than those with ≤10 years of experience (p=0.024). Furthermore, each one-point increase in knowledge score was associated with a 92% increase in the likelihood of exhibiting good clinical practice (p<0.001). The regression model was statistically significant (p<0.001) and explained 48% of the variance in clinical practice outcomes, indicating that qualification, experience, and knowledge are important determinants of restorative material selection practices for anterior teeth.

**Table 6:** Binary logistic regression analysis of factors associated with good clinical practice Binary logistic regression analysis was performed to identify factors associated with good clinical practice (practice score >75%). Data are presented as unstandardized regression coefficient (B), standard error (SE), Wald statistic, adjusted odds ratio (AOR), and 95% confidence interval (CI). A p-value of <0.05 was considered statistically significant. *Statistically significant predictor (p<0.05). Model fit statistics: χ²(3)=68.42; p<0.001; Nagelkerke R²=0.48. NSDP: non-specialist dental practitioner; SDP: specialist dental practitioner; df: degrees of freedom

Factor	B	SE	Wald	df	P-value	AOR	95% CI for AOR
Qualification (SDP vs. NSDP)	1.62	0.42	14.87	1	<0.001*	5.05	2.21-11.55
Years of experience (>10 years vs. ≤10 years)	0.88	0.39	5.09	1	0.024*	2.41	1.12-5.18
Knowledge score (per unit increase)	0.65	0.14	21.53	1	<0.001*	1.92	1.46-2.52

## Discussion

The present study evaluated the knowledge and clinical practice patterns of dental practitioners regarding restorative material selection for anterior teeth and compared the outcomes between NSDPs and SDPs. The findings demonstrated that SDPs possessed significantly higher knowledge scores, superior clinical practice patterns, and greater overall competency than NSDPs did. Professional qualifications, years of clinical experience, and knowledge level emerged as significant predictors of evidence-based restorative material selection.

Appropriate selection of restorative materials is fundamental to achieving optimal esthetic, functional, and biological outcomes in anterior restorative dentistry [6]. In the present study, SDPs demonstrated significantly higher knowledge scores than NSDPs. This finding is consistent with the observations of Kontakou Zoniou et al. [6], who reported that dentists with advanced education and specialty training exhibited greater familiarity with material properties, indications, and evidence-based restorative protocols. Advanced postgraduate training exposes specialists to contemporary literature, structured treatment planning approaches, and hands-on clinical experience, with a broader range of restorative materials, which may explain their superior performance. Similarly, previous surveys in restorative dentistry have reported that continuing professional education and specialization are associated with greater awareness of adhesive protocols, esthetic treatment planning, and biomaterial selection [7,8].

The present study also demonstrated significantly better clinical practice scores among SDPs. This finding suggests that higher theoretical knowledge translates into more evidence-based clinical decision-making. Specialists are more likely to select conservative restorative options for minimally damaged anterior teeth, employ appropriate adhesive strategies, utilize rubber dam isolation, and consider the current literature when planning treatment [9]. Similar observations have been reported in studies evaluating restorative decision-making, which have shown that clinicians with advanced training are more likely to adhere to contemporary minimally invasive dentistry principles [10,11]. The increasing emphasis on preserving a healthy tooth structure and utilizing adhesive restorative approaches may contribute to the differences between specialists and general practitioners.

Notably, only approximately one-third of participants demonstrated good knowledge and overall competency. Although restorative dentistry has undergone substantial technological advancements over the past two decades, the rapid introduction of new materials and restorative systems may create challenges for practitioners attempting to maintain current evidence-based recommendations [11]. Variability in access to continuing dental education programs, differences in clinical exposure, and reliance on personal experience rather than scientific evidence may contribute to these knowledge gaps. Similar concerns have been highlighted in previous investigations, which reported considerable variation in restorative material selection among practicing dentists despite the availability of clinical guidelines [12,13].

Correlation analysis revealed a strong positive association between the knowledge and practice scores. This finding indicates that practitioners with greater theoretical understanding are more likely to implement appropriate clinical practices. Comparable relationships have been reported in studies assessing professional competence among dental practitioners, where knowledge has been identified as a key determinant of evidence-based clinical behavior [6,14]. The results support the concept that educational interventions aimed at improving knowledge may directly influence clinical decision-making and treatment outcomes.

Years of clinical experience also demonstrated positive correlations with both knowledge and practice scores. Practitioners with more experience may have encountered a wider range of clinical situations, restorative failures, and long-term treatment outcomes, thus enabling more informed material selection. However, experience alone may not guarantee optimal practice, unless it is accompanied by continuous professional development. Several previous studies have suggested that experienced clinicians who actively participate in continuing education programs demonstrate superior evidence-based decision-making compared with practitioners who rely solely on traditional practice patterns [6,7,14,15].

Logistic regression analysis identified specialist qualification as the strongest predictor of good clinical practice. SDPs exhibited approximately five times greater odds of demonstrating evidence-based restorative decision-making than NSDPs. Additionally, higher knowledge scores independently increased the likelihood of good clinical practices. These findings emphasize the importance of postgraduate education and lifelong learning in restorative dentistry. The results also support previous reports indicating that educational attainment and professional training are significant determinants of treatment quality and adherence to contemporary restorative principles [16,17].

Another important observation was the significant influence of knowledge on clinical practice, independent of experience and qualification. This suggests that educational initiatives targeting knowledge enhancement may improve restorative decision-making, even among practitioners without specialty training. Structured continuing dental education programs, workshops on contemporary restorative materials, and the dissemination of evidence-based clinical guidelines may therefore play a crucial role in improving treatment standards [18,19].

Clinical implications

Our findings have several important clinical implications. The significant association between knowledge and clinical practice highlights the need for continuous professional education to ensure evidence-based restorative material selection for the anterior teeth. Dental institutions, professional organizations, and regulatory bodies should promote regular training programs focusing on contemporary restorative materials, adhesive protocols, esthetic treatment planning, and minimally invasive dentistry. Improving practitioner knowledge may enhance treatment quality, restoration longevity, patient satisfaction, and preservation of natural tooth structure.

Limitations

Several limitations of this study should be considered when interpreting these findings. First, its cross-sectional design precludes the establishment of causal relationships between knowledge and clinical practices. Second, the use of a self-reported questionnaire may introduce response and social desirability biases, potentially resulting in the overestimation of evidence-based practices. Third, convenience sampling may have limited the generalizability of our findings to all dental practitioners. Fourth, the actual clinical performance was not directly evaluated, and the responses may not fully reflect real-world clinical behavior. Finally, regional differences in educational opportunities, clinical exposure, and access to restorative materials were not specifically investigated. Future multicenter studies involving larger and more diverse populations combined with objective assessments of clinical decision-making are recommended to validate and expand the present findings.

## Conclusions

The present study demonstrated significant differences in knowledge and clinical practice patterns related to restorative material selection for anterior teeth between SDPs and NSDPs. Specialists exhibited higher knowledge scores, more evidence-based clinical practice patterns, and greater overall competency. Knowledge level, professional qualifications, and clinical experience were significantly associated with evidence-based restorative decision-making. These findings highlight the need for continued professional education and evidence-based training to support restorative treatment planning among dental practitioners. However, given the cross-sectional design of the study, these associations should not be interpreted as causal, and further longitudinal or interventional studies are warranted to determine whether educational interventions improve clinical practice and patient outcomes.

## Appendices

**Table 7:** Questionnaire used in the study

Demographic details	
Age	□ <30 years □ 30-40 years □ 41-50 years □ >50 years
Gender	□ Male □ Female
Qualification	□ BDS □ MDS
Specialty	□ Conservative Dentistry and Endodontics □ Prosthodontics □ Orthodontics □ Periodontics □ Oral Surgery □ Oral Medicine □ Pedodontics □ Public Health Dentistry □ Other __________
Years of clinical experience	□ <5 years □ 5-10 years □ 11-20 years □ >20 years
Type of practice	□ Private practice □ Academic institution □ Hospital-based practice □ Both academic and private practice
Knowledge-based questions	
Questions	Options
For a Class IV fracture involving less than one-third of the incisal edge in a young adult, the most conservative restorative option is?	□ Ceramic veneer □ Full ceramic crown □ Direct composite restoration □ Metal ceramic crown
Which restorative material requires the maximum removal of sound tooth structure?	□ Direct composite □ Porcelain veneer □ Full coverage ceramic crown □ Glass ionomer cement
The most important advantage of direct composite restorations in anterior teeth is?	□ High cost □ Conservative tooth preparation □ Superior wear resistance to ceramics □ No need for shade matching
For an anterior tooth with severe intrinsic discoloration and intact enamel, the preferred treatment is?	□ Composite restoration □ Ceramic veneer □ Glass ionomer restoration □ Temporary crown
The success of bonded anterior composite restorations primarily depends on?	□ Patient age □ Enamel bonding quality □ Gender □ Tooth position
Which material generally exhibits the best long-term color stability?	□ Glass ionomer cement □ Direct composite □ Ceramic restoration □ Zinc phosphate cement
Which factor most influences shade selection?	□ Room temperature □ Ambient lighting conditions □ Patient occupation □ Tooth number
The most suitable restoration for a tooth with >50% coronal structure loss is?	□ Direct composite □ Microfilled composite □ Full coverage crown □ Sealant
Which material demonstrates the highest translucency and esthetics?	□ Amalgam □ Glass ionomer □ Lithium disilicate ceramic □ Temporary acrylic
Rubber dam isolation during anterior adhesive restorations primarily improves?	□ Patient speech □ Moisture control and bond strength □ Shade selection □ Occlusal adjustment
Practice-based questions	
For a young patient with a small Class III lesion in a maxillary incisor, your preferred restoration is?	□ Direct composite □ Porcelain veneer □ Full ceramic crown □ Metal ceramic crown
How often do you use rubber dam isolation for anterior composite restorations?	□ Always □ Often □ Sometimes □ Never
For diastema closure in anterior teeth, your first treatment choice is?	□ Direct composite bonding □ Extraction □ Full crown □ Temporary restoration
When selecting restorative material, which factor is most important in your routine practice?	□ Patient esthetic demand □ Dentist preference only □ Manufacturer advertisement □ Availability of stock only
How do you usually perform shade selection?	□ Visual shade guide only □ Visual shade guide under natural light □ Random selection □ Laboratory determination only
For a severely discolored anterior tooth, your preferred restorative option is?	□ Composite restoration □ Ceramic veneer □ Temporary crown □ Glass ionomer restoration
How frequently do you discuss restorative material options with patients before treatment?	□ Always □ Often □ Sometimes □ Never
For fractured anterior teeth involving more than half of the crown structure, your preferred treatment is?	□ Direct composite restoration □ Composite veneer □ Full coverage crown □ Glass ionomer restoration
How often do you attend continuing dental education programs related to esthetic restorative materials?	□ At least once per year □ Once every 2-3 years □ Rarely □ Never
When restoring anterior teeth, how frequently do you consider evidence from current literature or clinical guidelines?	□ Always □ Often □ Sometimes □ Never
Knowledge score (0-10): poor knowledge: 0-4; moderate knowledge: 5-7; good knowledge: 8-10	
Practice score (0-10): For practice questions, evidence-based responses marked (*) receive 1 point. Poor practice: 0-4; moderate practice: 5-7; good practice: 8-10	
Total score (0-20): poor overall competency: 0-9; moderate overall competency: 10-14; good overall competency: 15-20	
